# Smoking and social inequalities in France, 20 years of evolution: A secondary dataset analysis of health barometers

**DOI:** 10.18332/tid/220422

**Published:** 2026-06-08

**Authors:** Anne Pasquereau, Romain Guignard, Raphaël Andler, François Beck, Viêt Nguyen-Thanh

**Affiliations:** 1 Sante Publique France, the French National Public Health Agency, Saint Maurice, France; 2 Centre de Recherche en Epidemiologie et Sante des Populations (CESP), Universite Paris-Saclay, Universite Paris-Sud, Universite Versailles Saint-Quentin, Villejuif, France

**Keywords:** tobacco smoking, inequalities, socio-economic status, tobacco control plans, France

## Abstract

**INTRODUCTION:**

Since the early 2000s, smoking prevalence has declined overall in France. This study aims to describe the evolution of social inequalities in smoking in relation to anti-smoking policy between 2000 and 2021.

**METHODS:**

This study is a secondary analysis of data from Santé Publique France's Health Barometers, cross-sectional telephone surveys conducted between 2000 and 2021 on random samples of the French population aged 18–75 years (between 9074 and 28224 people were surveyed, depending on the edition). Evolutions in prevalence according to socio-economic status (level of education, income, and occupational status) were modeled using Poisson regression.

**RESULTS:**

Overall, social inequalities related to smoking increased between 2000 and 2021, with the prevalence of daily smoking rising among individuals with lower levels of education (from 30% to 32%), lower incomes or unemployed, while declining among more advantaged groups (e.g. from 28% to 17% for individuals with the highest levels of education). The analysis reveals three distinct phases: an increase in inequalities between 2000 and 2016, e.g. the association between smoking and a level of education below high school increased (adjusted interaction term, AIT=1.027; 95% CI: 1.021–1.033, per year) compared to those with a level of education above high school. Disparities then stabilized between 2016 and 2019. Finally, a resumption of the increase in inequalities related to education and income was observed between 2019 and 2021, e.g. over time, the association between smoking and a level of education below high school increased (AIT=1.071; 95% CI: 1.001–1.141).

**CONCLUSIONS:**

The 2016–2019 period was marked by the first national tobacco control plan, including numerous measures that addressed social inequalities. Over the same period, smoking declined among all socio-economic groups, and differences in smoking prevalence according to socio-economic status have stabilized, bringing an end to 16 years of increase. Unfortunately, inequality began to rise again in 2020–2021, during the COVID-19 pandemic.

## INTRODUCTION

**Figure 1:**
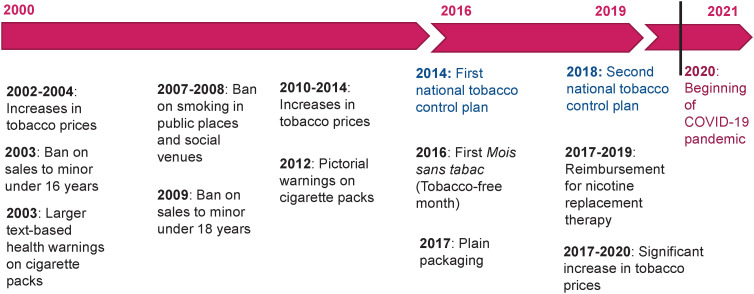
Measures implemented as part of France’s tobacco control policy, 2000–2021

Tobacco consumption is the leading cause of preventable death in France. In 2015, it was responsible for 75000 deaths, accounting for 13% of all fatalities^1^. In 2021, almost one in three French adults aged 18–75 years (32%) reported smoking, while a quarter (25%) said they smoked daily^2^. Since the early 2000s, the prevalence of daily smoking has evolved in three stages: a relative stability with levels close to 30% between 2000 and 2016, a marked decline between 2016 and 2019, followed by a stagnation between 2019 and 2021, probably linked to the COVID-19 crisis^2^, but also to less dynamic public policies (e.g. small price increases)^3^. The decline observed between 2016 and 2019 follows a particularly proactive public policy to combat smoking, with the implementation of national plans starting in 2014 (Figure 1). In particular, these plans led to the introduction of four measures that were considered effective or promising based on previous studies: the *‘Mois sans tabac’* (Tobacco-free month) social marketing campaign launched in 2016, inspired by the British ‘Stoptober’ campaign^4^, the introduction of plain packaging in 2017^5^, reimbursement for nicotine replacement therapy improved between 2017 and 2019^6^ and a significant increase in tobacco prices between 2017 and 2020^7^. The Organization for Economic Co-operation and Development (OECD) has estimated that these measures would be offset by savings in healthcare spending by 2050, providing an average return of €4 for every €1 invested^8^.

Analyses of overall smoking trends in the general population need to be supplemented by analyses of sub-populations, since different groups are affected by smoking in different ways^9^. Since the early 2000s, socio-economically disadvantaged populations have been significantly more likely to smoke, potentially due to specific motivations^2^. Socio-economically vulnerable groups are more likely to see cigarettes as a way of managing everyday stress^10^. They find it more difficult to plan for the future and place greater value on the present moment^11^. They dismiss the consequences of their behavior on their health in the medium- and long-term, and they even appear to deny the risks associated with tobacco use^12^. In addition, social norms tend to be more favorable towards smoking in environments where people are most disadvantaged, where a strong sense of belonging to the group can be quite strong^10,13^. Finally, disadvantaged populations tend to be less receptive to prevention messages. They are generally more skeptical, have lower literacy skills, and are critical of anti-smoking campaigns and communications issued by health authorities^10^.

Despite differences depending on the period and country concerned, overall, less privileged smokers are just as likely to want to quit as more privileged smokers. However, they may find it more difficult to quit^13^. They are also less likely to plan to quit in the short-term, less likely to attempt to quit smoking^14^, and their chances of successfully quitting are lower than for the more privileged^15^.

This article aims to describe evolutions in social inequalities in smoking in France since the early 2000s, in light of the anti-smoking policy implemented during this period.

## METHODS

### Data

This study is a secondary analysis of data from Santé Publique France’s Health Barometers, a survey measuring health behaviors conducted regularly between 2000 and 2021 (and even annually from 2016 to 2021). The survey covers people living in ordinary households in mainland France who speak French, aged 18–75 or 18–85 years, depending on the edition. The method is based on the random generation of landline and mobile phone numbers^16^. Participants are selected using a two-stage sampling on landlines (random selection of one eligible individual per household) and a single-stage sampling on mobile phones (selection of the person who answers the phone).

Estimates are weighted to take into account the probability of inclusion, based on telephone equipment and household size, as well as the population structure, via margin calibration using the following variables: gender crossed with age in ten-year brackets, region, urban unit size, household size and level of education (reference populations: National Institute of Statistics and Economic Studies, INSEE employment survey).

The analyses cover the age group of 18–75 years, which is common to all editions of the Barometers. The selected editions are: 2000, 2005, 2010, 2014, 2016, 2017, 2018, 2019, 2020, and 2021^17^.

### Variables

Smoking status was obtained with three questions: ‘Do you smoke tobacco, even if only occasionally?’. Those who answered ‘yes’ to the first question were then asked: ‘Do you smoke every day?’ and ‘How many cigarettes (manufactured or roll-your-own)/cigars/cigarillos/hookahs do you smoke on average?’. Respondents who reported either that they smoked every day (second question) or that they smoked a certain number of cigarettes or other tobacco products per day (third question) were classified as daily smokers.

Sociodemographic data collected in the surveys included gender and age. Level of education was collected with the question ‘What is the highest degree you have obtained?’ and recoded in three categories: <high school diploma, high school diploma, >high school diploma. Occupational status was collected with the question ‘What is your current employment status?’ and recoded into three categories: working, unemployed, student, or inactive. Household income was collected with the question ‘What is the total monthly net income for everyone in your household, including benefits?’. Household income was calculated per consumption units (CU) to compare households of both different sizes and compositions by assigning a coefficient to each member of the household: one CU for the first adult in the household, 0.5 CU for other people aged ≥14 years, and 0.3 CU for children under 14 years. Income was divided into tertiles based on the distribution observed in the sample: the first tercile for the third of the population with the lowest income, the second for the middle third, and the third for the third with the highest income. The full questionnaires are available online^17^.

### Statistical analysis

The sample was first described using frequencies and weighted percentages for each variable studied, along with 95% confidence intervals (95% CIs): gender, age group, education level, income and occupational status. Weighted percentages and 95% CI of daily smokers were also computed for each edition of the Health Barometer between 2000 and 2021.

The evolution of daily smoking prevalence according to the three socio-economic variables, level of education, income, and occupational status, was described with weighted percentage and 95% CI between 2000 and 2021. These three variables were selected because they are independently associated with daily smoking in France and are consistently included in the Barometer^2^.

To test the significance of the trends, a Poisson regression was performed for each subpopulation (e.g. those with the lowest level of education), with daily smoking as the dependent variable and year as the continuous explanatory variable, adjusting for gender and age. The adjusted prevalence ratio (APR) for the year variable (continuous) is presented with 95% CI for each of the 9 subpopulations (each of the three categories of the three socio-economic variables). As the evolution between 2000 and 2021 was not linear, three models were developed, each corresponding to one of the three periods identified in the trend description: 2000–2016, 2019–2021, and 2026–2019.

Sensitivity analyses were performed by adjusting each model for the other two socio-economic variables. For example, for the model among individuals without a high school diploma or with a diploma below the high school level, it was adjusted for income and employment status, in addition to gender and age. Any differences in the results are mentioned in the text.

Finally, the evolution of social inequalities in smoking was analyzed. To test the significance of trends in differences between the least and most advantaged groups, Poisson regressions were performed for each socio-economic variable, with daily smoking as the outcome and the interaction between the socio-economic variable and time (year, continuous) as the explanatory variable. The model was adjusted for gender and age, and the adjusted interaction terms (AIT) and 95% confidence intervals, were calculated. The interaction term represents the evolution in the relative gap between the least and most advantaged groups over time. A model was created for each of the three identified periods.

Sensitivity analyses were performed by adjusting each model for the other two socio-economic variables. For example, in the model examining the interaction between education level and year, income and employment status were added in addition to gender and age. Any differences in the results are mentioned in the text. The analyses were conducted using Stata software 16.0, and all tests were performed at a 95% significance level.

## RESULTS

### Sample description

Over the course of the 10 editions of the Health Barometer between 2000 and 2021, 176261 people aged 18–75 years were surveyed (Table 1). More than half (51.8%) had no diploma or a diploma below high school level, 19.5% had a high school diploma, and 28.7% had a diploma above high school level. More than half (56.0%) were employed, 8.9% were unemployed, and 35.0% were students or economically inactive. Between 2000 and 2016, the prevalence of daily smoking among those aged 18–75 years remained generally stable at around 30%. It then declined sharply to 24.0% in 2019, before stabilizing between 2019 and 2021 at around 25% (Table 2).

**Table 1:** Description of the study population aged 18–75 years in France, Health Barometer surveys, 2000–2021 (N=176261)

*Characteristics*	*Unweighted* *sample size* *n*	*Weighted* *percentages* *% (95% CI)*
**Gender**		
Male	79789	48.7 (48.4–49.0)
Female	96472	51.3 (51.0–51.6)
**Age** (years)		
18–24	17362	11.7 (11.4–11.9)
25–34	29646	17.5 (17.3–17.8)
35–44	32986	18.9 (18.6–19.1)
45–54	33773	19.4 (19.2–19.7)
55–64	34051	17.5 (17.3–17.7)
65–75	28443	15.1 (14.8–15.3)
**Education level**		
<High school diploma or no diploma	73330	51.8 (51.5–52.1)
High school diploma	35537	19.5 (19.2–19.7)
>High school diploma	67118	28.7 (28.5–29.0)
**Income**		
1st tercile (low)	46687	35.7 (35.4–36.1)
2nd tercile	56616	33.6 (33.3–33.9)
3rd tercile (high)	5891	30.6 (30.4–30.9)
**Occupational status**		
Working	101839	56.0 (55.7–56.3)
Unemployed	12751	8.9 (8.7–9.1)
Student/inactive	61671	35.0 (34.7–35.3)

**Table 2:** Sample sizes of Health Barometer surveys 2000–2021 and prevalence of daily smoking by year among adults aged 18–75 years in France (N=176261)

*Year*	*Unweighted sample size* *n*	*Weighted percentages* *% (95% CI)*
2000	12588	30.0 (29.0–30.9)
2005	28224	27.5 (26.9–28.2)
2010	25034	29.7 (29.1–30.4)
2014	15186	28.5 (27.6–29.5)
2016	14875	29.4 (28.4–30.4)
2017	25319	26.9 (26.2–27.6)
2018	9074	25.4 (24.3–26.6)
2019	9611	24.0 (22.9–25.1)
2020	13725	25.5 (24.5–26.5)
2021	22625	25.3 (24.5–26.1)

**Figure 2:**
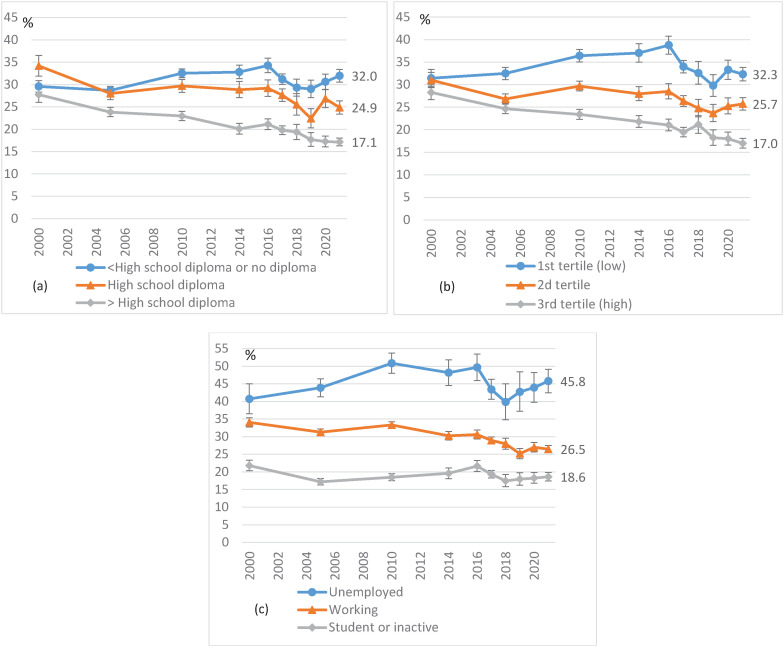
Prevalence of daily smoking by education level, income level, and occupational status among adults aged 18-75 years in France, Health Barometer surveys, 2000-2021 (N varying by wave)

Between 2000 and 2021, the prevalence of daily smoking among those aged 18–75 years according to level of education, income, and occupational status, evolved in three stages (Figure 2).

While prevalence remained stable overall in France between 2000 and 2016, different trends were observed according to three socio-economic variables. Prevalence increased among the least advantaged, while it decreased among the most advantaged. For instance, it rose from 31% to 39% in the lowest income group and fell from 28% to 21% in the highest income group.

Between 2016 and 2019, prevalence declined significantly across all populations. For example, there was a drop from 34% to 29% among those with the lowest levels of education and from 21% to 18% among those with the highest levels of education.

Finally, between 2019 and 2021, prevalence stabilized overall during the COVID-19 crisis. However, an increase can be observed among the most disadvantaged, which is significant among those with the lowest levels of education (from 29% to 32%).

### Poisson regressions

Between 2000 and 2016, a significant increase in daily smoking was observed among people with the lowest levels of education (APR=1.015; 95% CI: 1.011–1.018, per year), the third of the population with the lowest incomes (APR=1.009; 95% CI: 1.005–1.013), and unemployed people (APR=1.010; 95% CI: 1.004–1.016). Conversely, a significant decrease in daily smoking was observed among those with a degree higher than a high school diploma (APR=0.986; 95% CI: 0.981–0.990) and a third of the population with the highest incomes (APR=0.990; 95% CI: 0.985–0.994) (Table 3).

**Table 3:** Poisson regression estimates of daily smoking prevalence among adults aged 18–75 years in France. For each subpopulation and by period: association with the year, Health Barometer surveys, 2000–2021 (N varying by wave)

*Variables*	*2000–2016* *APR (95% CI)*	*2016–2019* *APR (95% CI)*	*2019–2021* *APR (95% CI)*
**Education level**			
<High school diploma or no diploma	**1.015 (1.011–1.018)**	**0.957 (0.933–0.981)**	**1.062 (1.022–1.104)**
High school diploma	0.995 (0.990–1.000)	**0.927 (0.895–0.959)**	1.045 (0.992–1.102)
>High school diploma	**0.986 (0.981–0.990)**	**0.947 (0.918–0.978)**	0.988 (0.941–1.037)
**Income**			
1st tercile (low)	**1.009 (1.005–1.013)**	**0.936 (0.910–0.964)**	1.037 (0.992–1.083)
2nd tercile	1.001 (0.997–1.005)	**0.947 (0.918–0.976)**	**1.051 (1.003–1.101)**
3rd tercile (high)	**0.990 (0.985–0.994)**	**0.954 (0.922–0.987)**	0.974 (0.922–1.028)
**Occupational status**			
Working	0.998 (0.995–1.000)	**0.939 (0.919–0.960)**	1.026 (0.992–1.061)
Unemployed	**1.010 (1.004–1.016)**	**0.943 (0.901–0.987)**	1.045 (0.974–1.121)
Student/inactive	1.004 (0.999–1.010)	**0.954 (0.919–0.991)**	1.024 (0.968–1.084)

APR: adjusted prevalence ratio. Between 2000 and 2016, among individuals with no diploma or a diploma lower than high school, an increase of one year was associated with an increase in the prevalence of daily smoking (APR=1.015; 95% CI: 1.011–1.018). Model adjusted for gender and age.

Between 2016 and 2019, there was a significant decline in daily smoking across all subpopulations.

Between 2019 and 2021, a significant upward trend emerged among individuals with lower levels of education (APR=1.062; 195% CI: 0.22–1.104) and middle incomes (APR=1.051; 95% CI: 1.003–1.101).

After adjusting each model for the other two socio-economic variables (sensitivity analysis), the trends observed in the main analysis remained unchanged. Three additional trends became significant: a decrease among high school graduates (APR=0.994; 95% CI: 0.989–0.999) and an increase among those with intermediate incomes (APR=1.004 ; 95% CI: 1.000–1.008) between 2000 and 2016; and an increase among those with the lowest incomes (APR=1.055; 95% CI: 1.010–1.101) between 2019 and 2021.

Between 2000 and 2016, the gap in daily smoking prevalence between the least and most advantaged groups increased significantly (Table 4). Over time, the association between smoking and a level of education below high school increased (adjusted interaction term AIT=1.027; 95% CI: 1.021–1.033, per year) and to a less extent for high school graduates (AIT=1.008; 95% CI: 1.001–1.015), compared to those with a level of education above high school. Similarly, the association between smoking and low income also increased over time (AIT=1.019; 95% CI: 1.013–1.025) and to a less extent for middle income (AIT=1.011; 95% CI: 1.005–1.017), compared to high income. Finally, the association between smoking and unemployment increased (AIT=1.014; 95% CI: 1.007–1.020) compared to being in employment.

**Table 4:** Poisson regression estimates of daily smoking prevalence among adults aged 18–75 years in France by period, according to socioeconomic variables: interaction between year and socio-economic level, Health Barometer surveys, 2000–2021 (N varying by wave)

*Variables*	*2000–2016* *AIT (95% CI)*	*2016–2019* *AIT (95% CI)*	*2019–2021* *AIT (95% CI)*
**Education level**			
<High school diploma or no diploma	**1.027 (1.021–1.033)**	1.008 (0.968–1.049)	**1.071 (1.001–1.141)**
High school diploma	**1.008 (1.001–1.015)**	0.978 (0.934–1.025)	1.054 (0.981–1.132)
> High school diploma (ref.)			
**Income**			
1st tercile (low)	**1.019 (1.013–1.025)**	0.980 (0.937–1.025)	1.067 (0.994–1.144)
2nd tercile	**1.011 (1.005–1.017)**	0.991 (0.947–1.037)	**1.080 (1.005–1.161)**
3 rd tercile (high) (ref.)			
**Occupational status**			
Working (ref.)			
Unemployed	**1.014 (1.007–1.020)**	1.003 (0.953–1.055)	1.017 (0.941–1.101)
Student/inactive	1.006 (0.999–1.012)	1.015 (0.972–1.061)	0.993 (0.930–1.060)

AIT: adjusted interaction term. Between 2000 and 2016, each additional year increases the prevalence ratio of daily smoking among individuals with less than a high school diploma or no diploma compared to those with a higher than high school diploma. A model for each of the three socio-economic variables, adjusted for gender and age.

Between 2016 and 2019, Poisson regression shows no significant evolution over time in the prevalence gaps between the least and most advantaged groups, according to the three socio-economic variables.

Between 2019 and 2021, the gaps widened again, but only among certain populations. Over time, the association between smoking and a level of education below high school increased (AIT=1.071; 95% CI: 1.001–1.141) compared to a level of education above high school. It was also the case for having middle incomes (AIT=1.080; 95% CI: 1.005–1.161) compared to having higher incomes.

After adjusting each model for the other two socio-economic variables (sensitivity analysis), the previously observed results remain unchanged. One additional significant trend emerges: between 2019 and 2021, the association between smoking and low income increases with each passing year (AIT=1.076; 95% CI: 1.004–1.153), compared to higher incomes.

## DISCUSSION

### Key findings

Between 2000 and 2016, social inequalities in smoking widened in France. The relative differences in daily smoking prevalence between the groups with the lowest and highest levels of education, income, and occupational status increased significantly. Between 2016 and 2019, social inequalities stabilized, with no significant evolution in smoking prevalence gaps. Finally, between 2019 and 2021, the gaps based on level of education and income increased again. The factors that may explain these evolutions differ across the three periods considered.

### Period 2000–2016

From the early 2000s, anti-smoking measures were gradually introduced as part of two Cancer Plans: bans on sales to minors (under 16 years in 2003 and under 18 years in 2009), increases in tobacco prices (2002–2004 and 2010–2014), a ban on smoking in public places and social venues (2007–2008), larger text-based health warnings on cigarette packets (2003) and then visual warnings (2012) (Figure 1). Smoking prevalence has declined among the most socially advantaged populations, who were the first to adopt prevention messages and policies. Disadvantaged populations took longer to grasp these messages and may have adopted strategies to avoid the increase in cigarette prices, such as switching to a cheaper product, rolling tobacco, or other means^18^. Finally, when the first cancer plan was being developed, the issue of social inequalities in smoking had only just begun to emerge, so tackling these inequalities was not a central concern.

### Period 2016–2019

The first two national tobacco control plans, implemented in 2014 and 2018, respectively, have been successful in reducing smoking prevalence and social inequalities in smoking. The simultaneous implementation of complementary measures has encouraged people to try to quit smoking, provided them with support and discouraged them from initiating smoking. In view of the disparities observed in the mid-2010s, particular attention was paid to tackling inequalities. For instance, the price of rolling tobacco was significantly increased to stop cigarette smokers switching to other products. Furthermore, sustained price increases can have a greater impact on the most disadvantaged^19^. The annual social marketing campaign, *Mois sans tabac* (Tobacco-free month), has also promoted the implementation of local initiatives targeting disadvantaged populations, increasing its impact among these groups^20^. Finally, the barriers to using smoking cessation aids have been partially removed owing to the reimbursement of nicotine replacement therapies by health insurance, effective 1 January 2019.

### Period 2019–2021

The COVID-19 pandemic has had psychological, economic, and social consequences, which have been more pronounced among disadvantaged populations^21^. Yet, among them, cigarettes can be more often perceived as a tool for managing stress or overcoming everyday difficulties^10,22^. Qualitative surveys conducted among smokers in 2020 and 2021 showed that smokers who perceived cigarettes as a tool for managing stress were more likely to increase their consumption^23^. The crisis may, therefore, have contributed to the increase in smoking prevalence among the most disadvantaged. In addition, tobacco shops remained open in France during lockdowns, as essential businesses, and messages claiming that nicotine protected against COVID circulated. The COVID-19 pandemic may also have indirectly impacted public anti-smoking policies by weakening prevention efforts. For instance, the 2020 ‘Tobacco-free month’ campaign was unable to be supported by local initiatives, and a decline in registrations and quit attempts was observed^24^. Finally, in 2020 and 2021, the price of tobacco rose only slightly, which deprived some smokers of a motivational lever to quit smoking^25^. Trends in social inequalities related to smoking in France following the COVID-19 crisis will be examined with future editions of the Health Barometer surveys, in light of the tobacco control measures implemented after the crisis.

### International comparisons

In countries where smoking prevalence has declined sharply, persistent and marked social inequalities are also evident. In the United Kingdom, for instance, smoking prevalence ranges from 6% among the most highly educated to 32% among those with no qualifications^26^. In the United States, prevalence ranges from 4% to 32% depending on level of education, and from 6% to 20% depending on income^27^.

Several European countries have studied trends in smoking-related inequalities. To assess how these trends relate to the implementation of tobacco control measures in different countries, the MPOWER framework (Monitor, Protect, Offer, Warn, Enforce, and Raise tax) – established by the WHO to help countries achieve their tobacco endgame goals – and its evolution between 2007 and 2022, can be used^28^. For European countries, an MPOWER score was calculated for the year 2020^29^. France, for its part, has implemented 88% of the MPOWER measures, with significant progress between 2016 and 2020. Several countries in Europe observed a widening of social inequalities in smoking in the early 2000s, as in France. Belgium saw its inequalities rise between 1997 and 2008^30^, and achieved 79% of MPOWER measures in 2020. Between 1986 and 2007, smoking prevalence increased among the most disadvantaged in one region of Spain, particularly among young adults, leading to greater inequality^31^. Spain then introduced further tobacco control measures from 2010 and again in 2018, reaching 89% of MPOWER measures by 2020. In the Netherlands, inequalities in smoking cessation and smoking initiation increased among women between 2001 and 2008^32^. The country increased the number of measures implemented from 2010 to 2014, reaching 65% by 2020. Between 2003 and 2012, inequalities related to education among men persisted and may even have widened in Germany^33^, which strengthened its anti-smoking measures in 2010 and 2014, reaching 67% of MPOWER measures by 2020. Inequalities in Italy increased between 1997 and 2010, particularly with regard to the initiation of smoking among young people^34^, which stepped up its anti-smoking efforts starting in 2016, but remained at 60% in 2020. Finally, a study of adolescents in 23 European countries revealed that inequalities based on parents’ socio-professional category increased in relation to most health-related behaviors, including tobacco use, between 2002 and 2014^35^. Following the implementation of additional tobacco control measures beginning in the 2010s in many European countries, it will be interesting to monitor the scientific literature on the future trends in social inequalities related to smoking in these countries, in order to assess whether these policies have had an impact on smoking-related inequalities.

### Strengths and limitations

Santé Publique France’s Health Barometer is based on a large, random sample of the French population. It allows robust analyses to be conducted, even within sub-populations. Significant work has been carried out to optimize the representativeness and quality of the data collected (survey method, interviewer training, etc.). The same methodology was used for the Barometers between 2000 and 2021, enabling evolutions in indicators to be tracked over time.

There are several limitations to the study. Firstly, Santé Publique France’s Health Barometer is based on self-reported data, which may introduce underreporting biases (e.g. social desirability or recall biases). However, these biases are likely to be small and relatively consistent in the context of observational surveys on tobacco use^36^. Secondly, the response rate to telephone surveys has been declining in France and internationally for several years. Those who refuse to respond to health surveys are more likely to engage in risky behaviors, which can affect the representativeness of the samples. Finally, given the repeated cross-sectional design, causal inferences cannot be drawn from the observed associations.

## CONCLUSIONS

Reducing social inequalities in smoking, which are prevalent in many Western countries, must be a key objective of public anti-smoking policies. Effective measures tend to be more effective among the most privileged, who are more likely to adopt them, and this can widen inequalities. Specific efforts are therefore needed to address this phenomenon and adapt tobacco control measures for disadvantaged populations. It is important to encourage disadvantaged smokers to make attempts to quit smoking, but it is also essential to better help them turn these attempts into successful cessation. Disadvantaged smokers still too often view quitting smoking as a solitary endeavor, the success of which depends solely on individual willpower^23^. One of the challenges is to encourage them to use proven aids: behavioral support, pharmacological treatments covered by health insurance, and free remote assistance. Healthcare professionals could also become more involved, as their support increases the chances of quitting smoking. Measures have been implemented with these goals in mind in France, and over the same period, between 2016 and 2019, inequalities were contained, with smoking declining among disadvantaged populations.

## Data Availability

The data supporting this research are available from the authors on reasonable request.

## References

[1] Bonaldi C, Boussac M, Nguyen-Thanh V. Estimation of the number of deaths attributable to smoking in France from 2000 to 2015. Bull Epidemiol Hebd. 2019;(15):278-84. https://beh.santepubliquefrance.fr/beh/2019/15/2019_15_2.html

[2] Pasquereau A, Andler R, Guignard R, et al. National and regional prevalence of smoking in France in 2021 among 18-75-year-olds, according to the Santé publique France health barometer. Bull Epidemiol Hebd. 2022(26):470-480. https://beh.santepubliquefrance.fr/beh/2022/26/2022_26_1.html

[3] Douchet MA. Smoking and quitting smoking in 2021. Observatoire français des drogues et des tendances addictives; 2022. Accessed April 14, 2026. https://www.ofdt.fr/sites/ofdt/files/2023-08/field_media_document-3327-doc_num--explnum_id-32755-.pdf

[4] Djian A, Guignard R, Gallopel-Morvan K, et al. From “Stoptober” to “Moi(s) Sans Tabac”: how to import a social marketing campaign. J Soc Mark. 2019;9(4):345-356. doi: 10.1108/JSOCM-07-2018-0068

[5] Hastings G B, Moodie C. Implementation and evaluation of the Australian tobacco plain packaging policy. Tob Control. 2015;24(Suppl 2). https://tobaccocontrol.bmj.com/content/tobaccocontrol/24/Suppl_2.toc.pdf

[6] Cadier B, Durand-Zaleski I, Thomas D, Chevreul K. Cost effectiveness of free access to smoking cessation treatment in France considering the economic burden of smoking-related diseases. PLoS One. 2016;11(2):e0148750. doi:10.1371/journal.pone.0148750 26909802 PMC4766094

[7] Chaloupka FJ, Yurekli A, Fong GT. Tobacco taxes as a tobacco control strategy. Tob Control. 2012;21(2):172-180. doi:10.1136/tobaccocontrol-2011-050417 22345242

[8] Devaux M, Dorfmuller Ciampi M, Guignard R, et al. Economic evaluation of the recent French tobacco control policy: a model-based approach. Tob Control. 2025;34(6):746-752. doi:10.1136/tc-2023-058568 39084903 PMC12703259

[9] U.S. Department of Health and Human Services. Centers for Disease Control and Prevention. National Center for Chronic Disease Prevention and Health Promotion. Office on Smoking and Health. Smoking Cessation: A Report of the Surgeon General; 2020. Accessed April 14, 2026. https://www.hhs.gov/sites/default/files/2020-cessation-sgr-full-report.pdf

[10] Peretti-Watel P, Constance J. “It’s all we got left”. Why poor smokers are less sensitive to cigarette price increases. Int J Environ Res Public Health. 2009;6(2):608-621. doi:10.3390/ijerph6020608 19440404 PMC2672354

[11] Peretti-Watel P, L’Haridon O, Seror V. Time preferences, socio-economic status and smokers’ behaviour, attitudes and risk awareness. Eur J Public Health. 2013;23(5):783-788. doi:10.1093/eurpub/cks189 23345320

[12] Institut National du Cancer. Tobacco and cancer: risk perception in 2021 and changes since 2015. January 30, 2023. Accessed April 14, 2026. https://www.cancer.fr/toutel-information-sur-les-cancers/comprendre-les-cancers/les-facteurs-de-risque/barometre-cancer/tabac-et-cancer

[13] Kotz D, West R. Explaining the social gradient in smoking cessation: it’s not in the trying, but in the succeeding. Tob Control. 2009;18(1):43-46. doi:10.1136/tc.2008.025981 18936053

[14] Guignard R, Soullier N, Pasquereau A, et al. Factors associated with the desire to quit smoking and quit attempts among smokers. Results of the 2021 Public Health France Barometer. Bull Epidemiol Hebd. 2023;(9-10):159-165. http://beh.santepubliquefrance.fr/beh/2023/9-10/2023_9-10_2.html

[15] Guignard R, Gaudebout A, Andler R, Pasquereau A, Beck F, Nguyen-Thanh V. Smoking cessation in metropolitan France: recent trends and associated factors based on data from the 2021 Public Health France Barometer. Bull Epidemiol Hebd. 2025;(10):172-80. https://beh.santepubliquefrance.fr/beh/2025/10/2025_10_2.html

[16] Richard J-B, Andler R, Gautier A, Guignard R, Leon C, Beck F. Effects of using an overlapping dual-frame design on estimates of health behaviors: a French general population telephone survey. J Surv Stat Methodol. 2017;5(2):254-274. doi:10.1093/jssam/smw028

[17] Baromètre. Public Health France Barometers. Santé publique France. Accessed April 14, 2026. https://www.santepubliquefrance.fr/etudes-et-enquetes/barometres-de-sante-publique-france

[18] Peretti-Watel P, L’haridon O, Seror V. Responses to increasing cigarette prices in France: how did persistent smokers react?. Health Policy. 2012;106(2):169-176. doi:10.1016/j.healthpol.2012.03.016 22502935

[19] Wilkinson AL, Scollo MM, Wakefield MA, Spittal MJ, Chaloupka FJ, Durkin SJ. Smoking prevalence following tobacco tax increases in Australia between 2001 and 2017: an interrupted time-series analysis. Lancet Public Health. 2019;4(12):e618-e627. doi:10.1016/S2468-2667(19)30203-8 31759897

[20] Guignard R, Andler R, Richard JB, et al. Effectiveness of ‘Mois sans tabac 2016’: a French social marketing campaign against smoking. Tob Induc Dis. 2021;19:60. doi:10.18332/tid/139028 34305506 PMC8288465

[21] Marsden J, Darke S, Hall W, et al. Mitigating and learning from the impact of COVID-19 infection on addictive disorders. Addiction. 2020;115(6):1007-1010. doi:10.1111/add.15080 32250482 PMC9364227

[22] Guignard R, Quatremère G, Pasquereau A, et al. Barriers against and motivations for quitting smoking during the COVID-19 health crisis: results of a qualitative study in France. Int J Environ Res Public Health. 2022;19(20):13051. doi:10.3390/ijerph192013051 36293628 PMC9602125

[23] Guignard R, Quatremère G, Pasquereau A, et al. Barriers against and motivations for quitting smoking during the COVID-19 health crisis: results of a qualitative study in France. Int J Environ Res Public Health. 2022;19(20):13051. doi:10.3390/ijerph192013051 36293628 PMC9602125

[24] Guignard R, Pasquereau A, Andler R, Avenel J, Beck F, Nguyen Thanh V. Effectiveness of the French Mois sans tabac on quit attempts in the first year of Covid-19: a population-based study. In: Prevention - Between Ethics and Effectiveness. Paper presented at: 13th EUSPR Conference and Members’ Meeting; September 28-30, 2022; Tallinn, Estonia. Accessed April 14, 2026. https://www.santepubliquefrance.fr/sites/default/files/rdd/document/565962_spf00004150.pdf

[25] Pasquereau A, Guignard R, Andler R, Nguyen Thanh V. Opinions on tobacco price increases and impact on motivation to quit in France. Tob Induc Dis. 2025;23(Suppl 1):A738. Paper presented at: World Conference on Tobacco Control; June 23-25, 2025; Dublin Ireland. Accessed April 14, 2026. https://www.tobaccoinduceddiseases.org/Opinions-on-tobacco-price-increases-and-impact-on-motivation-to-quit-in-France,206802,0,2.html

[26] Adult smoking habits in the UK: 2023. Office for National Statistics. Accessed April 14, 2026. https://www.ons.gov.uk/peoplepopulationandcommunity/healthandsocialcare/healthandlifeexpectancies/bulletins/adultsmokinghabitsingreatbritain/2023

[27] U.S. Centers for Disease Control and Prevention. 2022 National Health Interview Survey (NHIS) Highlights. Tobacco Product Use Among Adults; 2022. Accessed April 14, 2026. https://www.cdc.gov/tobacco/media/pdfs/2024/09/cdc-osh-ncis-data-report-508.pdf

[28] The pathway to a tobacco-free world. An interactive guide to the 2025 global tobacco epidemic report. World Health Organization. Accessed April 14, 2026. https://www.mpowerportal.org/

[29] González-Marrón A, Koprivnikar H, Tisza J, et al. Tobacco endgame in the WHO European region: feasibility in light of current tobacco control status. Tob Induc Dis. 2023;21:151. doi:10.18332/tid/174360 38026503 PMC10647070

[30] González-Marrón A, Koprivnikar H, Tisza J, et al. Tobacco endgame in the WHO European region: feasibility in light of current tobacco control status. Tob Induc Dis. 2023;21(November):151. doi:10.18332/tid/174360 38026503 PMC10647070

[31] Bacigalupe A, Esnaola S, Martín U, Borrell C. Two decades of inequalities in smoking prevalence, initiation and cessation in a southern European region: 1986-2007. Eur J Public Health. 2013;23(4):552-558. doi:10.1093/eurpub/cks104 22874737

[32] Nagelhout GE, de Korte-de Boer D, Kunst AE, et al. Trends in socio-economic inequalities in smoking prevalence, consumption, initiation and cessation between 2001 and 2008 in the Netherlands. Findings from a national population survey. BMC Public Health. 2012;12:303. doi:10.1186/1471-2458-12-303 22537139 PMC3356226

[33] Hoebel J, Kuntz B, Kroll LE, et al. Trends in absolute and relative educational inequalities in adult smoking since the early 2000s: the case of Germany. Nicotine Tob Res. 2018;20(3):295-302. doi:10.1093/ntr/ntx087 28431153

[34] Verlato G, Accordini S, Nguyen G, et al. Socio-economic inequalities in smoking habits are still increasing in Italy. BMC Public Health. 2014;14:879. doi:10.1186/1471-2458-14-879 25159912 PMC4159540

[35] Dierckens M, Richter M, Moor I, et al. Trends in material and non-material inequalities in adolescent health and health behaviours: a 12-year study in 23 European countries. Prev Med. 2022;157:107018. doi:10.1016/j.ypmed.2022.107018. 35283161

[36] Wong SL, Shields M, Leatherdale S, Malaison E, Hammond D. Assessment of validity of self-reported smoking status. Health Rep. 2012;23(1):47-53. 22590805

